# Prediction of the potency of mammalian cyclooxygenase inhibitors with ensemble proteochemometric modeling

**DOI:** 10.1186/s13321-014-0049-z

**Published:** 2015-01-16

**Authors:** Isidro Cortes-Ciriano, Daniel S Murrell, Gerard JP van Westen, Andreas Bender, Thérèse E Malliavin

**Affiliations:** Département de Biologie Structurale et Chimie, Institut Pasteur, Unité de Bioinformatique Structurale; CNRS UMR 3825, 25, rue du Dr Roux, Paris, 75015 France; Centre for Molecular Science Informatics, Department of Chemistry, University of Cambridge, Cambridge, UK; European Molecular Biology Laboratory European Bioinformatics Institute Wellcome Trust Genome Campus, Hinxton, Cambridge, CB10 1SD UK

**Keywords:** Proteochemometrics, Cyclooxygenases, Chemogenomics, QSAR, Ensemble modeling, Applicability domain

## Abstract

Cyclooxygenases (COX) are present in the body in two isoforms, namely: COX-1, constitutively expressed, and COX-2, induced in physiopathological conditions such as cancer or chronic inflammation. The inhibition of COX with non-steroideal anti-inflammatory drugs (NSAIDs) is the most widely used treatment for chronic inflammation despite the adverse effects associated to prolonged NSAIDs intake. Although selective COX-2 inhibition has been shown not to palliate all adverse effects (*e.g.* cardiotoxicity), there are still niche populations which can benefit from selective COX-2 inhibition. Thus, capitalizing on bioactivity data from both isoforms simultaneously would contribute to develop COX inhibitors with better safety profiles. We applied ensemble proteochemometric modeling (PCM) for the prediction of the potency of 3,228 distinct COX inhibitors on 11 mammalian cyclooxygenases. Ensemble PCM models ($R_{0\ test}^{2}=0.65$, and RMSE_test_ = 0.71) outperformed models exclusively trained on compound ($R_{0\ test}^{2}=0.17$, and RMSE_test_ = 1.09) or protein descriptors ($R_{0\ test}^{2}=0.16$ and RMSE_test_ = 1.10) on the test set. Moreover, PCM predicted COX potency for 1,086 selective and non-selective COX inhibitors with $R_{0\ test}^{2}=0.59$ and RMSE_test_ = 0.76. These values are in agreement with the maximum and minimum achievable $R_{0\ test}^{2}$ and RMSE_test_ values of approximately 0.68 for both metrics. Confidence intervals for individual predictions were calculated from the standard deviation of the predictions from the individual models composing the ensembles. Finally, two substructure analysis pipelines singled out chemical substructures implicated in both potency and selectivity in agreement with the literature.

**Electronic supplementary material:**

The online version of this article (doi:10.1186/s13321-014-0049-z) contains supplementary material, which is available to authorized users.

**Graphical Abstract:**
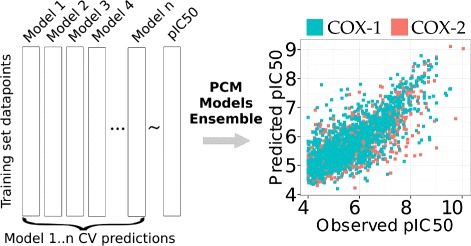
Prediction of uncorrelated bioactivity profiles for mammalian COX inhibitors with Ensemble Proteochemometric Modeling.

## Background

Cyclooxygenases (EC 1.14.99.1), also known as endoperoxidases, prostaglandin G/H synthases or simply COX, are involved in the biosynthesis of prostaglandin H_2_ from arachidonic acid [1]. Prostaglandin H_2_ is further converted into prostanoids which play a key role in inflammation. Thus, since the development of aspirin®; in 1899 [2], the inhibition of the cyclooxygenase activity with non-steroidal anti-inflammatory drugs (NSAIDs) has been exploited to treat inflammation. Nonetheless, kidney failure and gastrointestinal side-effects, such as peptic ulcer, have been correlated to long-term intake of NSAIDs [3]. Until 1991, only one form of the enzyme (COX-1) was thought to be responsible for both the constitutive and the local biosynthesis of prostaglandins. In that year [4], an inducible cyclooxygenase (COX-2) was discovered and the different roles of both isoenzymes were revealed. There does exist however some overlap: COX-1 is constitutively expressed serving as the source of housekeeping prostaglandins, whereas the expression of COX-2 increases in pathophysiological situations such as acute pain, inflammation or cancer [5]. From this it is thought that efficacy and side-effects can, to some extent, be delineated when blocking the prostaglandin synthesis pathway associated with inflammation and pain.

In the last two decades, research in both the pharmaceutical industry and academic laboratories has been driven by the hypothesis that selective COX-2 inhibitors would exhibit strong anti-inflammatory and analgesic properties without leading to the unwanted gastrointestinal side effects [6]. Nevertheless, a few organs, *e.g.* the brain cortex and renal glomeruli, express COX-2 constitutively [1]. The association between the inhibition of COX-2 in these organs with cardiovascular hazard (CVH) was ratified in 2004 and 2005 [7,8]. These findings led the US Food and Drug Agency (FDA) to retrieve rofecoxib (Vioxx) and valdecoxib (Bextra) from the market, and to include boxed warnings for all selective COX-2 inhibitors. Higher risk of heart attack and hypertension have also been reported for non-selective NSAIDs, thus highlighting that cardiovascular risk might not be related to the degree of COX selectivity [9]. In 2012, Yu *et al.* [10] demonstrated that the cardiovascular risk originates from COX-2 inhibition by selective and not selective NSAIDs and is taking place in blood vessels. These authors have shown that COX-2 inhibition leads to a decrease in prostaglandin (mainly PGI2) and to increased nitric oxide (NO) production which is sufficient to increase the risk of heart failure, hypertension and thrombosis [10].

Nevertheless, there are still niche populations which can benefit from selective COX-2 inhibitors, *e.g.* patients who cannot afford to take non-selective COX inhibitors, due to an increased risk of peptic ulcers or cancer. In addition, selective COX-2 inhibitors continue to be the common treatment for chronic inflammatory and pain disorders [3,11], and NSAIDs are known to reduce the risk of (among others) [12-15]: colon cancer [16-19], Alzheimer’s disease, and platelet aggregation [5,20]. Overall, NSAIDs are still one of the most commonly prescribed drugs in the world [21], and this trend is likely to increase owing to the aging of the population. Therefore, the administration of NSAIDs in clinics is currently subject to a benefit-risk assessment between the patients clinical profile and potential drugs side-effects [22], always aiming at optimizing both the dosage and the duration of the drug regimen [3].

The isoform selectivity of COX inhibitors stems from a structural difference in the binding site. The binding site of both cyclooxygenases is highly conserved except for the substitution of an isoleucine at position 523 in COX-1 with a valine in COX-2 [23]. This substitution results in a larger binding site in COX-2, as the smaller size of valine allows access to a side-pocket. This structural difference has been exploited for the rational design of potent and selective COX-2 inhibitors by both medicinal and computational chemistry [23-25]. To date, a plethora of *in silico* studies have been published with the aim of better understanding and predicting the potency of COX inhibitors on either COX-1 or COX-2 using molecular docking and QSAR models [26-30]. Nonetheless, none of these studies was able to integrate bioactivity information from multiple mammalian COX in the frame of a single machine learning model. Given that the bioactivity profiles of selective COX inhibitors on COX-1 and COX-2 are highly uncorrelated, thus presenting high selectivity ratios [24,25], only a predictive model trained on both the chemical and the target space would be able to simultaneously predict compound potency on a panel of cyclooxygenases, as well as to predict the activity of a given compound on a yet untested isoform. In that way, new potent, selective and safe COX inhibitors could be discovered.

Proteochemometrics (PCM) constitutes as an approach capable to simultaneously relate the chemical and the target space in single machine learning models in order to predict the bioactivity for a set of compounds against a panel of (related) biomolecular targets [31-33]. This integration of chemical and biological information enables, within the limits of the data presented to the model, the inter- and extrapolation on both the chemical and the target spaces to predict the potency of (novel) compounds on a panel of (novel) targets.

Therefore, the bioactivity of new compounds on yet untested targets can be predicted. These features of PCM make it different from both chemogenomics and QSAR, thus allowing [34,35]: (i) the inclusion of bioactivity information from orthologuous targets [34], (ii) bioactivity prediction for emergent viral mutations [35], or (iii) the design of personalized medicine for *e.g.* cancer treatment [33].

In this contribution, we apply the principles of PCM to model the potency of 3,228 compounds on 11 mammalian cyclooxygenases. To this aim, we have trained PCM models with different machine learning algorithms on public IC_50_ values from ChEMBL 16 [36], including data on human COX-1, COX-2, and on 9 orthologues. In an attempt to increase model performance, these models have been combined in ensembles (ensemble modeling), thus constituting the first PCM study where ensemble PCM modeling is applied. Additionally, the description of compounds with keyed fingerprints has enabled the deconvolution of the chemical space to rationalize both the potency and the selectivity of COX inhibitors towards a particular isoenzyme.

## Materials and methods

### Dataset

IC_50_ values for 11 mammalian cyclooxygenases, listed in Table 1, were retrieved from ChEMBL 16 [36]. To ensure the reliability of the bioactivity values, only IC_50_ values corresponding to small molecules and satisfying the following criteria were kept: (i) activity relationship equal to ‘ =’, (ii) assay score confidence ≥ 8, and (iii) activity unit equal to ‘nM’. The average pIC50 value was calculated when multiple IC_50_ values were annotated on the same compound-target combination. The application of these filters led to a final dataset composed of 3,228 distinct compounds and 11 sequences, being the total number of datapoints 4,937 (13.9% matrix completeness). The negative logarithm with base 10 of the IC_50_ values (pIC_50_) was used as the response variable to train all models. We decided to mix bioactivity data from different assays given that Kalliokoski *et al.* [37] reported that the standard deviation of public IC_50_ data is 25% larger than the standard deviation corresponding to public K_i_ data, and thus mixing IC_50_ data from different assays adds a moderate level of noise. The crystallographic structure of the ovine COX-1 complexed with celecoxib (PDB [38] ID: 3KK6 [39]) was used to extract the residues in the binding site. Those residues within a sphere of radius equal to 10Å centered in the ligand were selected.

**Table 1:** **Composition of the COX dataset**

**UniProt ID**	**Type**	**Organism**	**Number of bioactivities**	**% Compounds annotated**
P23219	1	*Homo sapiens*	1,346	41.7
O62664	1	*Box taurus*	48	1.5
P22437	1	*Mus musculus*	50	1.5
O97554	1	*Oryctolagus cuniculus*	11	0.3
P05979	1	*Ovis aries*	442	13.7
Q63921	1	*Rattus Norvegicus*	23	0.7
P35354	2	*Homo sapiens*	2,311	71.6
O62698	2	*Bos taurus*	21	0.7
Q05769	2	*Mus musculus*	305	9.4
P79208	2	*Ovis aries*	341	10.6
P35355	2	*Rattus Norvegicus*	39	1.2

The total number of bioactivities, after duplicate removal and selected from ChEMBL as described in Materials and methods, and of distinct compounds are 4,937 and 3,228 respectively. The last column indicates the percentage of the total number of distinct compounds (3,228) annotated on each target.

The corresponding residues for the other 10 sequences were identified by multiple sequence alignment [40]. The sequence alignment as well as the final residue selection are provided in the supplementary information.

### Computational details

### Descriptors

Chemical structures were standardized with the function *StandardiseMolecules* from the R package *camb* [41] with the following options: (i) inorganic molecules were removed, and (ii) molecules were selected irrespectively of the number of fluorines, chlorines, bromines or iodines present in their structure, or of their molecular mass. Morgan fingerprints [42,43] were calculated using RDkit (release version 2013.03.02) [44,45]. For the calculation of unhashed Morgan fingerprints [45], each compound substructure in the dataset, with a maximal diameter of four bonds, was assigned to an unambiguous identifier. Subsequently, substructures were mapped into an unhashed (keyed) array of counts. Physicochemical descriptors (PaDEL) [46] were calculated with the function *GeneratePadelDescriptors* from the R package *camb*. The R package *vegan* was used to generate the distributions of pairwise compound similarities (Jaccard distance) [47].

The amino acids composing the binding site of the mammalian cyclooxygenases considered in this study (Table 1), were described with five amino acid extended principal property scales (5 z-scales) [48]. Z-scales were calculated with the R package *camb* [41].

### Machine learning implementation

Machine learning models were built in the R statistical programming language using the packages *caret* [49] and *camb* [41]. Model ensembles were created with the help of the R package *caretEnsemble* [50]. Both the dataset (Additional file 1) and the modeling pipeline coded in R is available in the documentation of the R package *camb* [41].

### Model generation

Descriptors with a variance close to zero were removed with the function *RemoveNearZeroVarianceFeatures* from the R package *camb* using a cut-off value equal to 30/1 [41,49,51]. Subsequently, the remaining descriptors were centered to zero mean and scaled to unit variance with the function *PreProcess* from the R package *camb*.

The values of the model parameters were optimized by grid search and 5-fold cross validation (CV) [52]. The whole dataset was split into 6 folds by stratified sampling of the pIC_50_ values. One fold, 1/6, constituted the test set. The remaining folds, 5/6, were used to optimize the values of the parameters in the following way. For each combination of parameters, a model was trained on 4 folds, and the values for the remaining fold were then predicted. This procedure was repeated 5 times, each time holding out a different fold. The values of the parameters exhibiting the lowest average RMSE value along the 5 folds was considered as optimal. Subsequently, a model was trained on the whole training set, 5/6, using the optimized values for the parameters. The predictive power of this model was assessed on the test set, 1/6. To significantly compare the quality of the modeling with different machine learning algorithms, the same folds were used to train all models.

Both single PCM models and model ensembles were used to predict the bioactivities for the test set, and their error in prediction compared. The bioactivity values corresponding to the datapoints in the test set were not considered when building neither the single PCM models not the model ensembles.

In order to assess whether merging the chemical and the target space in a single PCM model enhances model performance, we trained two Random Forest (RF) models using either: (i) only compound descriptors (Family Quantitative Structure-Activity Relationship -QSAR-) [53], or (ii) only target descriptors (Family Quantitative Sequence-Activity Modeling -QSAM-) [53]. Obtaining a high performance with a Family QSAR model would indicate that the bioactivities of a given compound on different targets are correlated. Thus, target descriptors would not contribute to increase model performance. On the other hand, high performance observed for a Family QSAM model would indicate that the bioactivity values only depend on the targets and not on the compounds, *i.e.* the bioactivities of a set of diverse compounds are correlated on a given target. In this case, compound descriptors would not be required to predict compounds affinity, as target descriptors alone would be sufficient.

### Model validation

Both internal and external validation were performed according to the criteria proposed by Tropsha *et al.* [54-56], and to the RMSE values (Equation 1). The formulae of the statistical metrics used in the internal $\left (\text {RMSE}_{\text {int}} \;\text {and}\; q_{\textit {int}}^{2}\right)$ and the external $\left (\text {RMSE}_{\text {test}}, q_{\textit {test}}^{2}\; \text {and}\; R_{test\ 0}^{2}\right)$ validation are:

(1) $$ RMSE = \sqrt { \frac {\left(y - \widetilde{y}\right)^{2}} {N} }  $$

(2) $$ q^{2} = 1 - \frac {\sum_{i=1}^{N} \left(y_{i} - \widetilde{y}_{i}\right)^{2}} {\sum_{i=1}^{N} \left(y_{i} - \bar{y}\right)^{2}}  $$

(3) $$ {R_{0}^{2}} = 1 - \frac {\sum_{i=1}^{N} \left(y_{i} - \widetilde{y}_{i}^{r0}\right)^{2}} {\sum_{i=1}^{N} \left(y_{i} - \bar{y}\right)^{2}}  $$

where *N* represents the size of the training or test set, *y*_*i*_ the observed bioactivity values, $\widetilde {y}_{i}$ the predicted bioactivity values, and $\bar {y}$ the average values of the response variable for those datapoints included into either the training or the test set, and $\widetilde {y}^{r0} = s \widetilde {y}$, with $s = \frac {\sum {y_{i} \widetilde {y}_{i}}} {\sum {\widetilde {y}_{i}^{2}}} $.

Generally, to consider a model as statistically sound, the statistical metrics must satisfy the following criteria: (i) $q_{\textit {int}}^{2} >$ 0.5, and (ii) $q_{\textit {test}}^{2}$ and $R_{test\ 0}^{2} >$ 0.6. $R_{test\ 0}^{2}$ imposes the regression line to pass through the origin (intercept equal to zero). Although we follow these requirements here, as they serve as general guidelines for the evaluation of model predictive ability [54-56], the cut-off values for $q_{\textit {int}}^{2}$, $q_{\textit {test}}^{2}$ and $R_{test\ 0}^{2}$ should be adjusted and tailored in other studies depending on the dataset to be modeled.

### Assessment of maximum model performance

To further assess the reliability of the models in the light of the uncertainty of the bioactivity values [37,57,58], we established the maximum $R^{2}_{0\ test}$ and $q^{2}_{\textit {test}}$, and minimum RMSE_test_ values achievable given: (i) the uncertainty (experimental error) of public IC_50_ data, and (ii) the number of datapoints in both the training and the test set. The distributions of minimum RMSE_test_, and maximum $q_{\textit {test}}^{2}$, and $R_{0\:test}^{2}$ values were calculated in the following way.

Firstly, a random sample, *A*, was generated from the pIC50 values with a size equal to the test set. Secondly, the sample *A*_*noisy*_ was calculated by adding to *A* random noise with mean zero and standard deviation equal to the experimental error. The experimental error required to define the random samples *A*_*noisy*_ was taken as 0.68 pIC_50_ unit, which corresponds to the average standard deviation value for public IC_50_ datasets, as estimated by Kalliokoski *et al.* [37] Then, the statistical metrics were calculated for *A* with respect to *A*_*noisy*_. These steps were repeated 1,000 times, which permitted to define the distributions for the statistical metrics.

The maximum and minimum values of respectively $R^{2}_{0\ test}/q^{2}_{\textit {test}}$ and RMSE_test_ were then used to validate model performance on the test set.

If the obtained metrics were beyond the maximum values (for $q_{\textit {test}}^{2}$ and $R_{0\:test}^{2}$) or the minimum values (for RMSE_test_) of the corresponding distributions, the model is likely to be over-optimistic [52]. This estimation of the maximum achievable model performance takes into account the range and distribution of the bioactivities present in the data. This is of particular importance as it has been recently reported by Sheridan [59] that (i) certain bioactivity ranges are better predicted than others, and (ii) ${R_{0}^{2}}$ values might be very low if the bioactivity range considered is too narrow, even if the predictions closely match the observed values.

### Ensemble modeling

Gradient-boosting machines (GBM) [60], Random Forest (RF) [61], and Support Vector Machines (SVM) [62] were implemented to train a model library. The resulting models were combined in model ensembles using two techniques, namely: greedy optimization and model stacking. Depending on the models considered when training an ensemble, two types of model ensembles were defined: (i) homo-ensembles: the same algorithm was used to train all models composing the ensemble, though the parameter values were different in each model, (ii) hetero-ensembles: the number of distinct algorithms used to train the models combined in the ensemble was greater or equal than 2.

#### Greedy optimization

Greedy optimization, based on the work of Caruana *et al.* [63], optimizes the RMSE on the cross-validation predictions on the hold-out folds. These predictions were calculated for all models in the model library. These models were trained on a training set with identical fold composition. Each model was assigned a weight in the following manner. Initially, all models had a weight equal to zero. Afterwards, the weight of a given model was repeatedly incremented by 1 if the subsequent normalized weight vector allowed a closer match between the weighted combination of cross-validated predictions and the observed pIC50 values. This repetition was carried out *n* times, *n*=1000 in the present work, and the resulting weight vector was normalized to obtain the final models weighting. The predicted activity for a given compound corresponds to the weighted sum (using the optimized model weight vector) of the predictions generated by the individual models. The final model ensemble was used to predict the activities on the test set, and the error in prediction compared to that of single PCM models on the same set.

#### Model stacking (MS)

**Figure 1:**
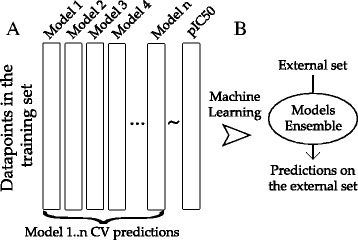
**Ensemble modeling with model stacking.** **A**. A set of models are trained with diverse machine learning algorithms (*Model1.. Model n* in the Figure). The predictions of these models on each datapoint in the training set calculated during cross validation, are used as descriptors to create a new training matrix, which rows are indexed by the datapoints in the training set and columns by the models in the library. A machine learning model is trained on this matrix. The resulting model is the model ensemble. **B**. The model ensemble is then applied on the test set.

The concept of model stacking is illustrated in Figure 1. In this case, the predictions on the training set calculated with the model library during cross-validation served as descriptors. Thus, a training matrix was defined where rows were indexed by the datapoints in the training set used to train the model library, and columns by the models in the aforesaid library.

A machine learning model was trained on this matrix, irrespective of the algorithms used to generate the model library. This model is then used to predict the bioactivities for the test set, and the RMSE value compared to that of single PCM models on the test set. To predict the activity for a compound from the test set, the individual models composing the ensemble are used to predict its activity (pIC50). These activities are then used as input features to the model stacking ensemble, which will output the predicted pIC50 value by the ensemble. The bioactivity values corresponding to the datapoints in the test set are not considered when building the ensemble. If the selected algorithm has the inherent capability to determine the importance of each descriptor, as for Elastic Net, a vector of weights for the models can be defined. Given that each descriptor corresponds to a particular model, this vector will determine its contribution to the generated ensemble. In the present study we used the following algorithms: linear model, Elastic Net, SVM with linear and radial kernels, and RF.

### Estimation of the error of individual predictions

In order to estimate errors for individual predictions, we used the standard deviation of the predictions of the individual models composing a given model ensemble, *i.e.* ensemble standard deviation (E_std_). Previous studies [59,64-66] have highlighted the usefulness of considering the ensemble standard deviation as a domain applicability (DA) measure, specially in the case of RF models, where the calculation of the standard deviation along the trees is straightforward [59,64]. Here, we extend this idea to ensembles composed of models trained with different algorithms (hetero-ensembles). For each datapoint in either the test set or in the hold-out fold in the case of cross-validation, we calculated the standard deviation of the predictions generated with each model conforming the model ensemble. Subsequently, the ensemble standard deviation was scaled with the parameter *β*. This permits to obtain individual confidence intervals for each prediction, which are thus defined as:

(4) $$ IC = \widetilde{y} \pm E_{std}\ \beta \ \left\{\beta \in \mathbb{R} \ | \ \beta >0\right\}  $$

To assess the practical usefulness of the derived confidence intervals, the percentage of datapoints for which the predicted values lied within *IC* (0<*β*<4) was calculated. Both the predictions calculated during model training (using the optimal parameter values), *i.e.* cross-validated predictions, as well as the predictions on the test set were used.

### Interpretation of compound substructures

**Figure 2:**
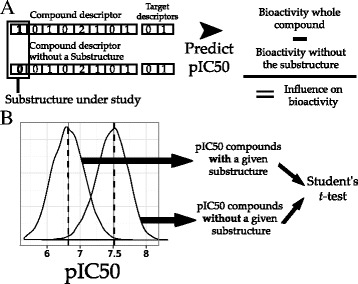
**Interpretation of compound substructures.** **A**. Predictive method. The average influence on bioactivity of a given substructure is calculated as the difference between the distributions corresponding to: (i) the predicted bioactivity for all compounds containing that substructure, and (ii) the predicted bioactivity using PCM for these compounds, from which that substructure was virtually removed by setting its count to zero. **B**. Student’s method. In this case, the average substructure influence on bioactivity is evaluated as the difference between the mean values of the pIC_50_ distributions for those compounds presenting and not presenting a given substructure. The statistical significance of this difference was evaluated with a Student’s t-test.

The contribution of chemical substructures to bioactivity on human cyclooxygenases was deconvoluted using a predictive and a Student’s method (Figure 2):

#### Prediction of bioactivity values with and without each compound substructure (predictive method)

This first technique quantifies the contribution of each chemical substructure to bioactivity by calculating the distribution of differences between (i) the predicted bioactivity for all compounds containing a given substructure, and (ii) the predicted bioactivity using PCM for these compounds, from which that substructure was virtually removed [67-72].

To virtually remove a substructure, we iteratively set its count equal to zero in all compound descriptors presenting it. The difference between the predicted bioactivity values in the presence or absence of a given substructure was then calculated. The average value of these differences, weighted by the number of counts of the feature in each compound, corresponds to the average contribution of that feature to bioactivity [68]. The contribution was estimated for all compound features considered in the model. The sign of the difference ({+/-}) indicates whether the feature is respectively beneficial or deleterious for compound bioactivity.

#### Statistical significance between bioactivity distributions with and without each compound substructure (Student’s method)

In order to identify chemical substructures that might not be recognized by the predictive method due to moderate PCM model performance, we also deconvoluted the chemical space in a model-independent way. We created two bioactivity sets, each containing the pIC_50_ values for either human COX-1 or human COX-2. For each of these bioactivity sets and for each substructure, we defined two distributions, namely:

(i) the distribution *A* of pIC_50_ values corresponding to the compounds presenting a given substructure in a given bioactivity set, and (ii) the distribution *B* of pIC_50_ values for those compounds not presenting that substructure in the same bioactivity set. The normality of these distributions was assessed with the Shapiro-Wilk test (*α*=0.05). If both distributions, *A* and *B*, followed the Gaussian distribution, a two-tailed *t*-test for independent samples (*α*=0.05) was applied to statistically evaluate the difference between them. If the difference was significant, we assumed that the considered substructure has an influence on bioactivity on the isoenzyme associated to the bioactivity set considered.

The sign of the difference between the mean value of *A* and *B* indicates whether the presence of the substructure hampers or fosters compound bioactivity on that isoenzyme. Therefore, each substructure was assigned a label, ‘deleterious’ or ‘beneficial’, depending on its influence on bioactivity on either COX-1 and COX-2.

Finally, we intended to assess which substructures always increase or decrease compound bioactivity on human COX-1 and COX-2. In that way, substructures identified in the previous step are finally identified as: (i) increasing or decreasing bioactivity on human COX-1, (ii) increasing or decreasing bioactivity on human COX-2, and (iii) increasing or decreasing bioactivity on both human COX-1 and COX-2.

## Results

### Analysis of the chemical and the target space

#### Target space

The PCA analysis of the amino acid descriptors of the binding site of the 11 mammalian cyclooxygenases (Table 1) is shown in Additional file 2: Figure S1. Orthologue sequences COX1 and COX2 define two distant clusters. As paralogues display more sequence variability than orthologues, and as small molecules tend to display similar binding within orthologues [73], we hypothesize that merging bioactivities from orthologues and paralogues will lead to more predictive models. In addition, these results indicate that the amino acid descriptors account for structural differences between COX-1 and COX-2.

#### Chemical space

The initial bioactivity selection from ChEMBL 16 [36], consisted of 6,804 datapoints. As previously highlighted [57], a large number of target-compound combinations in ChEMBL are annotated with more than one bioactivity value, hence the total number of different compound-target combinations after duplicate removal was 4,937.

The standard deviations for the bioactivity values annotated on the same compound-target combination are in less than 2% of the cases higher than two pIC_50_ units (Additional file 3: Figure S2A), whereas more than 90% of the repeated bioactivities exhibit a standard deviation close to zero (Additional file 3: Figure S2B). Consequently, we decided to take the average of these repeated values instead of the median value: this latter value would be more suitable only if outliers were more aboundant.

#### Selectivity dataset

**Figure 3:**
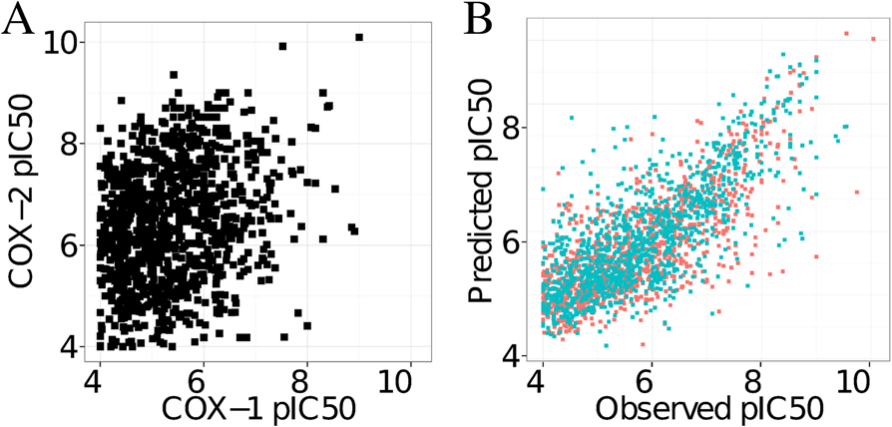
**COX inhibitors selectivity on human COX-1 and COX-2.** **A**. Scatterplot corresponding to the comparison of bioactivities against human COX-1 and COX-2 for 1,288 compounds. A large proportion of the compounds present a COX-2/COX-1 selectivity ratio between 2 and 4 pIC_50_ units. Therefore, the present dataset includes COX inhibitors with highly divergent bioactivity profiles for COX-1 and COX-2 (${R^{2}_{0}} = -0.42$). **B**. Scatterplot of the observed against the predicted pIC_50_ values for the compounds described in **A**. Blue squares correspond to the activity on COX-1, whereas orange squares correspond to the activity on COX-2. The PCM models explain more than 59% of the variance (${R^{2}_{0}} = 0.59$), thus highlighting the ability of the PCM models to predict the potency of compounds displaying uncorrelated bioactivity profiles on human cyclooxygenases.

As stated in the introduction, the main advantage of a PCM model applied to mammalian cyclooxygenases would be to anticipate the potency of a given compound towards a particular isoenzyme. To ensure that our dataset covered chemical entities with diverse bioactivity profiles on COX-1 and COX-2, we selected all compounds annotated on both human cyclooxygenases. This resulted in a selection of 1,086 compounds, out of a total of 3,228 distinct inhibitors present in the dataset. The scatterplot of the bioactivities of these compounds on human COX-1 against human COX-2 (Figure 3A) reveals that the difference in bioactivity for some compounds depending on the isoenzyme is higher than 4 pIC_50_ units (upper left corner of Figure 3A). RMSE and ${R_{0}^{2}}$ values for the bioactivities on COX-1 with respect to COX-2 are, respectively, 1.69 pIC_50_ units and -0.42. As the area above the diagonal of Figure 3A is more populated, there are more compounds with higher activity on COX-2 than on COX-1. Therefore, these data let us conclude that the dataset comprises compounds exhibiting high selectivity towards COX-2. In addition, the overlap between the datapoints in the PCA of the compound descriptors (Additional file 4: Figure S3) indicates that the compounds annotated on the COX targets cover the same regions of the chemical space.

### PCM validation

**Figure 4:**
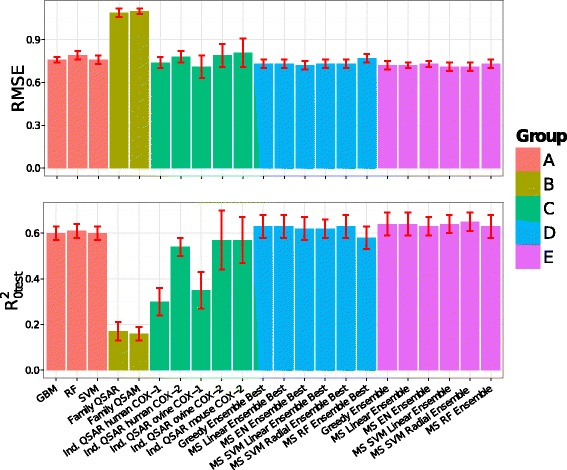
**Model performance on the test set.** RMSE_test_ (upper panel) and $R^{2}_{0\ test}$ (lower panel) values for the following models: (group A) single PCM, (group B) Family QSAR and Family QSAM, (group C) individual QSAR, (group D) model ensembles comprising those single PCM models exhibiting the highest predictive power, and (group E) model ensembles comprising the whole model library. Bars are colored according to the groups defined in Table 2. Confidence intervals correspond to the mean value +/- one standard deviation calculated with bootstrapping [74].

Overall, the models obtained with GBM, RF, and SVM (Table 2A and Figure 4) satisfied our model validation criteria, described in Materials and methods (Equations (1) to (3)), namely: $q_{\textit {int}}^{2} >$ 0.5 and, $q_{\textit {test}}^{2}$ and $R_{test\ 0}^{2} >$ 0.6. The performance of the three algorithms is comparable since $R^{2}_{0\ test}$ values range from 0.60 to 0.61, and RMSE_test_ from 0.76 to 0.79 pIC_50_ units between the different models. Interestingly, the predictive power did not vary when using hashed or unhashed fingerprints, being the $R^{2}_{0\ test}$ and RMSE_test_ differences smaller than 0.01 in both cases (data not shown). Thus, we decided to rather use unhashed fingerprints as this choice enables an interpretation of the models according to chemical substructures.

**Table 2:** **Internal and external validation metrics (mean values +/- one stardard deviation) for the PCM (A), Family QSAM (B), Family QSAR (B), Individual QSAR models (C), Ensemble PCM models combining the most predictive models (D), and Ensemble PCM models combining the whole model library (E)**

		${q^{2}_{\textit {int}}}$	**RMSE**_**int**_	${R^{2}_{0\ test}}$	**RMSE**_**test**_	${q^{2}_{\textit {test}}}$	**CCC**
A	GBM	0.59 +/- 0.02	0.77 +/- 0.01	0.60 +/- 0.03	0.76 +/- 0.02	0.60 +/- 0.03	0.76 +/- 0.02
	RF	0.60 +/- 0.03	0.78 +/- 0.02	0.61 +/- 0.03	0.79 +/- 0.03	0.61 +/- 0.03	0.74 +/- 0.02
	SVM	0.61 +/- 0.03	0.75 +/- 0.03	0.60 +/- 0.03	0.76 +/- 0.03	0.60 +/- 0.03	0.76 +/- 0.02
B	Family QSAR	0.17 +/- 0.02	1.13 +/- 0.02	0.17 +/- 0.04	1.09 +/- 0.03	0.17 +/- 0.04	0.43 +/- 0.03
	Family QSAM	0.16 +/- 0.02	1.10 +/- 0.02	0.16 +/- 0.03	1.10 +/- 0.02	0.16 +/- 0.03	0.28 +/- 0.02
C	Ind. QSAR human COX-1	0.31 +/- 0.04	0.75 +/- 0.05	0.30 +/- 0.06	0.74 +/- 0.04	0.30 +/- 0.06	0.45 +/- 0.05
	Ind. QSAR human COX-2	0.60 +/- 0.24	0.78 +/- 0.03	0.54 +/- 0.04	0.78 +/- 0.04	0.53 +/- 0.04	0.68 +/- 0.03
	Ind. QSAR ovine COX-1	0.28 +/- 0.11	0.83 +/- 0.08	0.35 +/- 0.08	0.71 +/- 0.08	0.09 +/- 0.09	0.50 +/- 0.07
	Ind. QSAR ovine COX-2	0.53 +/- 0.07	0.78 +/- 0.06	0.57 +/- 0.13	0.79 +/- 0.08	0.57 +/- 0.13	0.74 +/- 0.09
	Ind. QSAR mouse COX-2	0.49 +/- 0.08	0.84 +/- 0.10	0.57 +/- 0.10	0.81 +/- 0.10	0.57 +/- 0.11	0.71 +/- 0.07
D	Greedy Ensemble Best	-	0.73 +/- 0.01	0.63 +/- 0.05	0.73 +/- 0.03	0.63 +/- 0.05	0.77 +/- 0.02
	MS Linear Ensemble Best	0.63 +/- 0.02	0.73 +/- 0.01	0.63 +/- 0.05	0.73 +/- 0.03	0.63 +/- 0.05	0.78 +/- 0.02
	MS EN Ensemble Best	0.63 +/- 0.02	0.72 +/- 0.02	0.62 +/- 0.05	0.72 +/- 0.03	0.62 +/- 0.05	0.78 +/- 0.02
	MS SVM Linear Ensemble Best	0.63 +/- 0.01	0.73 +/- 0.02	0.62 +/- 0.04	0.73 +/- 0.03	0.63 +/- 0.05	0.78 +/- 0.02
	MS SVM Radial Ensemble Best	0.63 +/- 0.02	0.73 +/- 0.02	0.63 +/- 0.05	0.73 +/- 0.03	0.63 +/- 0.05	0.78 +/- 0.02
	MS RF Ensemble Best	0.61 +/- 0.01	0.76 +/- 0.01	0.58 +/- 0.05	0.77 +/- 0.03	0.58 +/- 0.05	0.75 +/- 0.02
E	Greedy Ensemble	-	0.73 +/- 0.01	0.64 +/- 0.05	0.72 +/- 0.03	0.64 +/- 0.05	0.78 +/- 0.02
	MS Linear Ensemble	0.63 +/- 0.02	0.73 +/- 0.02	0.64 +/- 0.05	0.72 +/- 0.02	0.64 +/- 0.05	0.78 +/- 0.02
	MS EN Ensemble	0.64 +/- 0.01	0.73 +/- 0.01	0.63 +/- 0.04	0.73 +/- 0.02	0.63 +/- 0.04	0.78 +/- 0.02
	MS SVM Linear Ensemble	0.64 +/- 0.03	0.73 +/- 0.04	0.64 +/- 0.04	0.71 +/- 0.03	0.64 +/- 0.04	0.80 +/- 0.02
	MS SVM Radial Ensemble	0.64 +/- 0.02	0.73 +/- 0.02	0.65 +/- 0.04	0.71 +/- 0.03	0.65 +/- 0.04	0.80 +/- 0.02
	MS RF Ensemble	0.64 +/- 0.02	0.73 +/- 0.02	0.63 +/- 0.05	0.73 +/- 0.03	0.63 +/- 0.05	0.78 +/- 0.02

“Best” refers to the ensembles trained on only the three most predictive RF, GBM and SVM models. MS of models trained with different algorithms in a models ensemble allows to increase predictive ability, as the highest $\textit {R}^{\text {2}}_{\text {0}\; \textit {test}}$ and RMSE_test_ values, 0.652 and 0.706 pIC _50_ units respectively, were obtaind with the “MS SVM Radial Ensemble”. The standard deviation for the metrics was calculated with the bootstrap method [74].

*Abreviations*: *CCC* Concordance Correlation Coefficient [75,76], *EN* Elastic Net, *GBM* Gradient Boosting Machine, *Ind.* Individual, *MS* Models Stacking, *RF* Random Forest, *RMSE* root mean square error in prediction, *SVM* Support Vector Machines.

To ensure that our modeling results did not arise from chance correlations, we trained models with an increasingly bigger fraction of the bioactivity values randomized (y-scrambling) [77]. The representation of model performance as a function of the percentage of randomized bioactivities is given in Additional file 5: Figure S4. When approximately 35% of the bioactivity values are randomized, $R^{2}_{0\ test}$ values become negative, which indicates that the relationships found by our models between both the chemical and the target space, and the bioactivity values are not spurious [77].

### PCM models are in agreement with the maximum achievable performance

**Figure 5:**
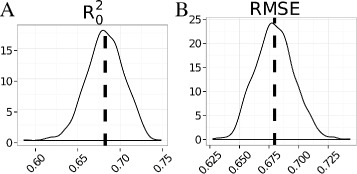
**Distribution of theoretical** $R_{0\ test}^{2}$ **(A) and RMSE**_**test**_ **(B) values.** The mean of the $R_{0\ test}^{2}$ distribution, 0.68, highlights that the uncertainty in public bioactivity data does not permit models displaying $R_{0\ test}^{2}$ values close to 1. Similar results were obtained for $q^{2}_{\textit {test}}$. From these data we conclude that the minimum RMSE_test_ value that a model can achieve without exhibiting overfitting is close to the experimental uncertainty.

The distributions of the respectively maximum and minimum achievable $R^{2}_{0\ test}$ and RMSE_test_ values are depicted in Figure 5. The maximum correlation values $R^{2}_{0\ test}$ are far from 1, which agrees with observations previously reported for public data [68,78]. The mean of the minimum theoretical RMSE_test_ values lies between 0.68 and 0.69, which is comparable to the level of uncertainty in public IC_50_ data reported by Kalliokoski *et al.* [37] The mean of the distribution of theoretical $R^{2}_{0\ test}$ values is between 0.67 and 0.69. The minimum RMSE_test_ and maximum $R^{2}_{0\ test}$ values obtained with the individual models, 0.76 and 0.61 respectively (Table 2A and Figure 4), thus appear consistent with the underlying uncertainty in the present dataset.

### PCM outperforms both family QSAR and family QSAM on this dataset

Interestingly, neither the Family QSAR nor the Family QSAM model alone could infer the relationships in the dataset, as the respective $R^{2}_{0\ test}$ and RMSE_test_ values were: (i) for Family QSAR: 0.17 and 1.09 pIC_50_ units, and (ii) for Family QSAM: 0.16 and 1.10 pIC_50_ units (Table 2B and Figure 4). Taken together, these results suggest that: (i) compound bioactivities on different targets are not correlated, as indicated by the low performance of the Family QSAR model, and (ii) compound bioactivities depend on compounds structure, as highlighted by the low performance of the QSAM model.

### PCM outperforms individual QSAR models

We then evaluated on individual targets the usefulness of PCM in comparison with QSAR models (Table 2C and Figure 4). Independent QSAR models for those targets with more than 100 bioactivities, namely: human COX-1 and COX-2, ovine COX-1 and COX-2, and mouse COX-2. The human COX-2 model exhibits a RMSE_test_ value of 0.78 pIC_50_ units, which is 0.03 pIC_50_ units larger than the RMSE_test_ value for the datapoints annotated on human COX-2 averaged over ten PCM models, namely 0.76 +/- 0.04 pIC_50_ units. By contrast, the $R^{2}_{0\ test}$ value drops to 0.54, indicating the higher peformance of PCM. Better correlations are obtained for the individual QSAR models corresponding to both the mouse and the ovine COX-2, for which the $R^{2}_{0\ test}$ values are 0.57 in both cases, whereas the RMSE_test_ values are 0.81 and 0.79 pIC_50_ unit. In contrast, the human and the ovine COX-1 QSAR models cannot relate the descriptor space to the bioactivity values in a statistically sound manner, as they exhibit respective $R^{2}_{0\ test}$ values of 0.30 and 0.36.

Altogether, these data evidence the versatility of PCM to integrate incomplete information from different protein targets. Furthermore, PCM strongly outperforms one-target and one-space models (Family QSAR, individual QSAR, and Family QSAM) [33].

### Model ensembles exhibit higher performance than single PCM models

As the most predictive PCM model exhibited moderately high $R^{2}_{0\ test}$ and $q^{2}_{\textit {test}}$ values, as well as moderately low RMSE_test_ values (Table 2A and Figure 4), we explored the possibility of enhancing model performance by combining different models into a more predictive model ensemble (Table 2D, E and Figure 4). Two ensemble techniques were implemented, namely: greedy optimization and model stacking (MS), previously described in section “Ensemble modeling”. To gather a library of diverse models, we trained a total of 282 GBM, RF and SVM models. Each of these models was trained with different parameter values. Hence, the performance of single models ranged from very poor to that of the individual models described above (Table 2A and Figure 4).

Initially, we created ensembles using only the most predictive GBM, RF and SVM models (Table 2D and Figure 4). Overall, all model ensembles (Table 2D) exhibited higher predictive power than single models (Table 2A). The best $R^{2}_{0\ test}$ value, 0.63, was obtained with the greedy and the MS linear ensemble. The weights for the three models in the greedy ensemble were: (i) GBM: 0.35, (ii) RF: 0.12, and (iii) SVM: 0.53. The MS Elastic Net ensemble displayed the highest predictive power, with a RMSE_test_ value of 0.72 (Table 2D and Figure 4). The small differences in performance observed between ensembles, with the exception of the RF ensemble are negligible, since, in the experience of the authors [68], the standard deviation observed for the $R^{2}_{0\ test}$ and RMSE_test_ values when using different samples during model training are between 0.1 and 0.3. The only model that led to worse results was the RF ensemble, with $R^{2}_{0\ test}$ and RMSE_test_ values of 0.58 and 0.77 pIC _50_ unit, respectively.

In a second step, ensembles were optimized using all models in the model library, namely 282 (Table 2E and Figure 4). Interestingly, the values of the statistical metrics for all ensembles increased.

The MS SVM ensemble with radial kernel displayed the highest predictive ability, with $R^{2}_{0\ test}$ and RMSE_test_ of 0.65 and 0.71 pIC_50_ unit, which only differs marginally from the minimum theoretical RMSE_test_ value, namely 0.68 (Figure 5).

**Figure 6:**
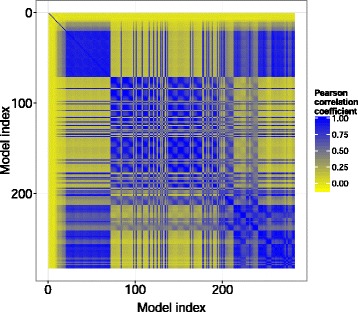
**Pairwise Pearson correlation for the cross-validation predictions across the model library.** The predictive power across the model library is not uniformly distributed, as the predicted values for a large fraction of model pairs are uncorrelated (yellow areas). Therefore, the combination of these models in a model ensemble is expected to lead to higher predictive power than individual models (“wisdom of crowds”).

Worthy of mention is the lack of performance improvement (data not shown) of homo-ensembles (*i.e* ensembles created with models trained with the same algorithm but with different parameter values) with respect to the most predictive single models (Table 2A and Figure 4), as the difference in $R^{2}_{0\ test}$ and RMSE_test_ values was below 0.01 for both metrics. By contrast, the ensembles exhibiting the highest predictive power on the test set were obtained when combining models with high and low predictive ability. This increase in performance is likely to arise from the fact that these models display uncorrelated resampling profiles, *i.e.* the predictions calculated on the hold-out folds during cross-validation are not correlated (Figure 6).

Overall, these data underline the highest predictive power of hetero-ensembles generated with a model library displaying a comprehensive range of predictive abilities.

### The ensemble standard deviation enables the definition of informative confidence intervals

**Figure 7:**
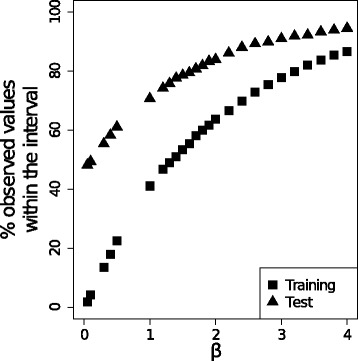
**Confidence intervals calculated from the ensemble standard deviation of the models present in the model ensembles.** The percentage of datapoints which predicted bioactivities lie within confidence intervals calculated with increasingly larger *β* values (Equation 4), is shown for: (i) the cross validated predictions calculated during model training (*Training* in the Figure), and (ii) for the predictions on the test set (*Test* in the Figure) calculated with the most predictive model ensemble, namely “Stacking SVM Radial Ensemble”. The percentage of true values lying within the confidence interval derived for a given *β* value increases with the number of datapoints available during model training. Overall, the confidence intervals derived from the ensemble standard deviation provide an estimation of the reliability of individual predictions, as in practice, this plot can be used to determine the *β* value corresponding to a given confidence level.

Figure 7 displays the percentage of datapoints which predicted values lie within confidence intervals calculated with increasingly larger *β* values (Equation 4). The ensemble model exhibiting the highest predictive power (RMSE_test_: 0.71; $R^{2}_{0\ test}$: 0.65), namely MS SVM Radial Ensemble, was used to make the predictions and to calculate the confidence intervals. Confidence intervals calculated for the cross-validated predictions (shown as squares in Figure 7) require larger *β* values to reach a given level of confidence when compared to those calculated on the test set (shown as triangles in Figure 7). This can be seen as the percentage of datapoints for which the true value is within the confidence interval (*β*=1) for the cross-validated predictions is 40%, whereas this value increases till 70% in the case of the test set. This difference might be due to the fact that predictions on the test set are made with models trained on a larger fraction of the dataset. Nevertheless, the error in prediction on the test set might increase if the compounds present therein were structurally dissimilar. In those cases, a larger *β* value would be required, with respect to that for the training set, to reach a given confidence level.

Overall, the percentage of true values lying within the confidence interval derived for a given *β* value is expected to increase with model performance. Figure 7 can be used to determine the *β* value corresponding to the confidence interval required by the user.

### Ensemble modeling enables the prediction of uncorrelated human COX inhibitor bioactivity profiles

As previously stated, selectivity is a crucial aspect in the discovery and optimization of COX inhibitors. To assess whether PCM models were able to predict the pIC_50_ values for compounds displaying uncorrelated bioactivity profiles on human COX-1 and COX-2, we predicted the bioactivity values for the 1,086 compounds annotated on both human COX-1 and COX-2. Figure 3B, which displays the observed against the predicted pIC_50_ values for these compounds, shows that PCM models are able to predict the potency for compounds displaying uncorrelated bioactivity profiles on human cyclooxygenases. Indeed, the $R^{2}_{0\ test}$ and RMSE_test_ values calculated for the observed pIC_50_ values with respect to those predicted by the PCM model are, respectively, 0.59 and 0.76 pIC_50_ unit.

Subsequently, we analyzed the capability of PCM models to correctly predict the bioactivity for both selective and non-selective compounds. A compound was considered as selective or non selective if the absolute value of the difference between its bioactivity on COX-1 and COX-2 is larger or smaller than 2 pIC_50_ units. On this basis, 226 compounds were considered as selective, and 860 as non selective. The error in prediction for the non selective compounds was lower than 1 pIC_50_ unit in 85.4% of the cases, and lower than 0.5 pIC_50_ unit for 55.6% thereof. On the other hand, the error in prediction was lower than 1 pIC_50_ unit for 73.23% of the selective compounds, and lower than 0.5 pIC_50_ unit for 42.9% thereof. When considering a more stringent selectivity cut-off value, namely 3 pIC_50_ units, we obtained a set of 61 compounds. The error in prediction for this set was lower than 1 pIC_50_ unit in 66.4% of the cases, and lower than 0.5 pIC_50_ unit for 40.2% thereof.

Consequently, these data indicate that PCM models are capable to predict the potency for both selective and non selective compounds on human COX-1 and COX-2. In addition, we anticipate that model performance is likely to increase with the inclusion of more bioactivity data in the models.

### Model performance *per* target is related to compound diversity

To further assess model performance on a *per* target basis, we generated 10 RF models each one trained on a different subset of the whole dataset.

The variation of performance across the 11 cyclooxygenases considered can be related to the compound diversity (Additional file 6: Figure S5).

Human cyclooxygenases, with the highest number of annotated compounds (Table 1), exhibited average RMSE_test_ values between 0.74 and 0.76 pIC_50_ unit. For these proteins, the distributions of pairwise compound similarity (Additional file 6: Figure S5) are skewed towards high similarity values, with mean values between 0.75 and 0.85.

**Figure 8:**
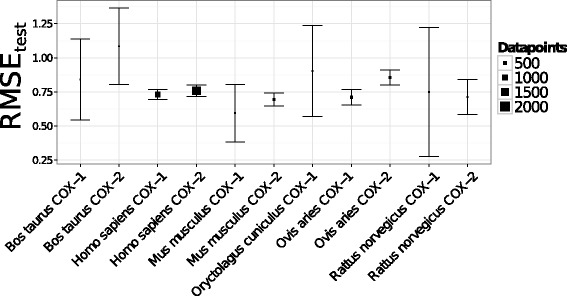
**Target-averaged model performance.** The number of datapoints is displayed through the size of the squares. Targets annotated with less than 30 compounds or with chemical structures displaying high structural diversity (*Oryctolagus cuniculus* COX-1, *Rattus norvegicus* COX-1, *Bos taurus* COX-1, and *Bos taurus*) are produced with high mean RMSE_test_ values. These observations indicate that PCM models are not always able to extrapolate in the chemical or the target space if a given target or compound family is not sufficiently represented in the dataset.

Likewise, mouse COX-2 and ovine COX-1 display average RMSE_test_ values of 0.70 and 0.73 pIC_50_ unit probably related to the smaller number of compounds annotated on these proteins (Table 1). High predictive ability on mouse COX-2 was expected given the high $R^{2}_{0\ test}$ value, 0.57, obtained with the individual QSAR model, whereas low performance was expected for ovine COX-1, as the individual QSAR model displayed a $R^{2}_{0\ test}$ value of 0.36. Unsurprisingly, skewed distributions in compound diversity are observed for mouse COX-2 and ovine COX-1 (Figure 8).

Conversely, ovine COX-2, with 341 annotated compounds, displayed a worse average RMSE_test_ value, within the 0.80-0.85 pIC _50_ unit range (Figure 8). This decrease in performance for ovine COX-2 might be ascribed to the higher dispersion of the pairwise compound similarity distribution with respect to those observed for mouse COX-2 and ovine COX-1 (Additional file 6: Figure S5).

The dependency of model performance on compound diversity is even more contrasted for targets with less than 100 annotated bioactivities. Indeed, the average RMSE_test_ value for mouse COX-1, with 50 compounds, lies within the 0.57-0.62 range of pIC_50_ unit and the distribution of compunds diversity is skewed towards high similarity values (Additional file 6: Figure S5). However, the average RMSE_test_ value increases till 0.80-0.90 pIC_50_ unit for bovine COX-1 (Additional file 6: Figure S5), annotated with 48 bioactivities and for which the pairwise compound similarity distribution presents several peaks, thus highlighting the structural diversity of the compounds. Finally, targets with less than 30 annotated compounds exhibit multimodal pairwise similarity distributions and, consequently, model performance is low, with standard deviations in the 0.50-1.00 range of pIC_50_ unit (Figure 8).

Overall, chemical diversity in the training set contributes to enhance the applicability of a PCM model. Nonetheless, a balance needs to be established between this diversity and the number of datapoints to ensure model convergence.

### Interpretation of compound substructures

#### Predictive method

The usage of unhashed fingerprints permitted the deconvolution of the chemical space to determine the influence of compound substructures on bioactivity. Two substructure analysis methodologies were implemented, as described in the section “Interpretation of Compound Substructures”. The first approach, predictive method, relies on the PCM model to correctly predict the bioactivity for a compound when a given substructure is virtually removed from a compound descriptor. The second approach, Student’s method, is a pipeline designed to statistically assess how the presence of a given substructure influences, on average, bioactivity on the compounds.

**Figure 9:**
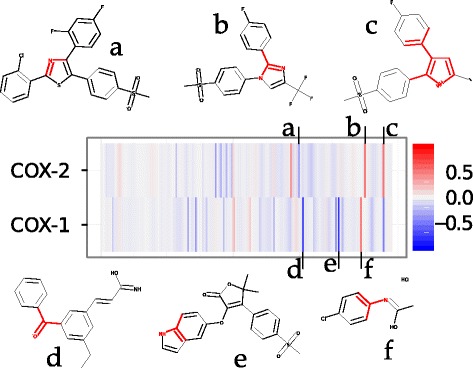
**Influence of compound substructures on potency and selectivity on human COX-1 and COX-2.** Rows in the heatmap are indexed by the isoenzyme type whereas columns correspond to compound substructures. Substructures are depicted in red within arbitrary molecules presenting it. The color represents the average influence (pIC_50_ units) of each substructure on bioactivity. Red corresponds to an average increase in bioactivity, whereas blue indicates a deleterious effect. Well-known chemical moieties, *e.g.* pyrrole rings (c), were singled out as selectivity determinants. For instance, substructure d is present in sulfonamides such as diflumidone, and substructure b in selective 1,2-diarylpyrroles COX-2 inhibitors.

Figure 9 shows the contribution to bioactivity of each substructure considered in the model on human COX-1 and COX-2 calculated with the predictive method. Red and blue areas correspond respectively to substructures that, on average, enhance or decrease compound bioactivity. Representative substructures either deleterious or beneficial for bioactivity are also shown. Generally, substructures shown to have an influence on bioactivity display an opposite behaviour depending on the isoenzyme type. For example, a pyrrole ring with aryl substituents in the 2,3-positions (substructure **c** in Figure 9) is predicted to have a high influence on bioactivity, increasing it on COX-2 and decreasing it on COX-1. This observation is in agreement with the literature as the 2,3-diarylpyrrole series with an halogen substituent in the 5-position acting as electron withdrawing group have been found as selective COX-2 inhibitors [79,80]. The pyrrole moiety with a radical in the 1-position is also found as a selectivity feature towards COX-2 (substructure **b** in Figure 9). This agrees with the discovery by Khanna *et al.* [81] of a series of 1,2-diarylpyrroles as potent and selective COX-2 inhibitors.

On the other hand, substructures conferring a deleterious effect could also be identified. substructure **e** in Figure 9 is represented within compound 3-(1H-indol-5-yloxy)-5,5-dimethyl-4-(4-methylsulfonylphenyl)furan-2-one (CHEMBL322276). This compound is part of a series of 3-heteroaryloxy-4-phenyl-2(5H)-furanones reported as selective COX-2 inhibitors by Lau *et al.* [82]. Its COX-1/COX-2 selectivity ratio is larger than 4.17, which agrees with the prediction of decreasing bioactivity on COX-1. In general, substructures decreasing bioactivity tend to be small and less informative (*e.g.* single atoms or substructures with two heavy atoms), than those fostering compound potency.

#### Student’s method

**Figure 10:**
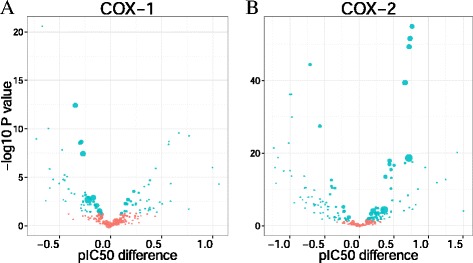
**Volcano plots corresponding to the results of the Student’s method applied on human COX-1 (A) and COX-2 (B).** The size of the points is proportional to the number of molecules in the dataset containing a given substructure. Significant *P* values are shown in red (two-tailed *t*-test, *α*=0.05).

The implementation of the Student’s method to deconvolute the chemical space (Figure 10), which evaluates the statistical significance between bioactivity distributions in the presence or absence of each compound substructure, led to the following observations: (i) 74 substructures increase bioactivity on COX-2, (ii) 64 substructures decrease bioactivity on COX-2, (iii) 9 substructures increase bioactivity on COX-1, (iv) 2 substructures decrease bioactivity on COX-1, (v) 1 substructure increases bioactivity on both COX-1 and COX-2, and (vi) 6 substructures decrease bioactivity on both COX-1 and COX-2.

Well-known chemical moieties conferring selectivity to COX-2 were present in this substructure selection. Additional file 7: Figure S6 shows the 20 substructures predicted to have the highest influence to increase bioactivity on human COX-2. For instance, substructures containing thiazole, pyrrole, pyrazole and oxazole rings were enriched for COX-2 [24,25]. Likewise, tri-fluorometil and sulfonamide radicals, which appear in *e.g.* celecoxib, were also enriched [24]. Substructures predicted to influence in the same way the compound bioactivity on both COX-1 and COX-2 are small, which makes difficult to extract medicinal chemistry knowledge therefrom (Additional file 8: Figure S7).

It is nevertheless remarkable that the output of both methods is contradictory for some substructures. By way of example, substructure **d** in Figure 9 is considered as deleterious for bioactivity on COX-1 by the predictive method, whereas it is regarded as beneficial by the Student’s method. Dannhardt *et al.* [83] highlighted the key role of the carbonyl moiety for the potency of a series of diarylmethanone compounds on both COX isoenzymes. Nonetheless, Scholz *et al.* [84] have recently reported a series of *ortho-*carbaborane derivatives of indomethacin as selective COX-2 inhibitors. Furthermore, substructure **d** also appears in a series of [2-[(4-substituted or 4,5-disubstituted)-pyridin-2-yl]carbonyl-(5- or 6-substituted or 5,6-disubstituted)-1H-indol-3-yl]acetic acid analogues identified as COX-2 inhibitors [85]. Plausible reasons for this divergence are analyzed in the Discussion section.

Overall, both substructure analysis pipelines have proven to be able to highlight chemical moieties conferring or decreasing potency and selectivity in agreement with the literature.

## Discussion

In this contribution two ensemble modeling techniques, namely greedy optimization and model stacking, have been presented and benchmarked on a PCM dataset comprising the bioactivities of COX inhibitors on 11 mammalian cyclooxygenases (Table 1). PCM has been shown to relate the target and the chemical spaces to bioactivity in a statistically sound manner (Table 2) [54-56]. Family QSAR as well as Family QSAM displayed poor performance (Table 2B and Figure 4).

Three machine learning algorithms (GBM, RF and SVM) have been implemented individually and combined in model ensembles. The application of ensemble modeling has been shown to outperform single machine learning models, the improvement being larger if the three most predictive GBM, RF and SVM models are combined in the same ensemble (Table 2D and Figure 4). Nonetheless, the model stacking (MS) SVM radial kernel model trained on the predictions of a library of 282 single PCM models (Table 2E and Figure 4) displayed the lowest RMSE_test_ and the highest $R^{2}_{0\ test}$ values. This non-linear model combination led to a RMSE_test_ value comparable to the experimental uncertainty of public pIC_50_ data [37]. It is noteworthy to mention that this ensemble was obtained by combining several hundreds of poor and highly predictive models instead of only the most predictive models of each class, namely GBM, RF and SVM (Table 2D and Figure 4). Therefore, these results suggest that if sufficient computing resources are available, higher predictive ability can be obtained with a large and diverse model library. Given that the ensemble concept is not restricted to any particular machine learning algorithm, the pipeline proposed in this study can be further explored.

The variability in the predictions of the individual models composing model ensembles, quantified by the ensemble standard deviation, served to define informative confidence intervals. Previous studies highlighted the usefulness of this variability as an applicability domain metric [59,64-66]. Here, we have extended this concept to ensembles of models trained on different algorithms (Figure 7). The higher performance of model ensembles has already been observed [86,87]. This phenomenon, usually termed ‘wisdom of crowds’, arises from the fact that different models provide complementary information. Moreover, the combination of a number of models palliates the effect of extreme predictions by averaging them (regression to the mean), and the chances of obtaining erroneous predicitons with a single model decrease. Interestingly, it has been recently reported in the context of cell line sensitivity prediction [87] that higher performance was obtained by combining moderate predictive models, instead of the most predictive models of each class. This observation has been corroborated in the present study (Table 2E and Figure 4). Overall, the application of ensemble modeling with a model library trained with either the same algorithm but different parameter values (homo-ensemble), or with different algorithms (hetero-ensemble) constitutes a promising alternative to single models in the context of predictive bioactivity modeling.

High predictive ability for compounds displaying uncorrelated bioactivity profiles on COX-1 and COX-2 was attained with both single models and model ensembles (Figure 3B). Therefore, the present study illustrates how the combination of the target and the chemical spaces in a single PCM model improves the prediction of compound potency in the context of multi-target systems. The implications of COX-2 in widespread diseases, *e.g.* cancer, has prompted the design of potent and selective COX-2 inhibitors since the early 1990s [24,25]. Thus, the suitability of PCM to predict COX inhibitor potency and to integrate multispecies bioactivity data opens new avenues for the design of cyclooxygenase inhibitors.

The two approaches presented in this study for the deconvolution of the chemical space, namely: (i) bioactivity prediction with and without a given compound substructure (predictive method), and (ii) assessment of the statistical difference between the bioactivity distributions corresponding to compounds presenting or not a given compound substructure (Student’s method), singled out chemical moieties responsible for COX-2 selectivity in agreement with the medicinal chemistry literature.

The divergent results described for substructure **d** in Figure 9, plausibly arise from the following properties of the two methods.

As in the predictive method the bioactivity is predicted by calculating the average difference between the predicted value for a compound with and without a given substructure, the (potentially non-linear) relationships between the substructures present in a molecule can be established, and the dependence of bioactivity on additional substructures or scaffolds present in the molecule accounted. On the other hand, the Student’s method considers the substructures as independent. The two methods can thus give contrasted results for example in the following case. We can envision a compound, *A*, presenting a substructure, *S*_1_, having no effect on bioactivity, and a second substructure, *S*_2_, strongly fostering bioactivity on the studied biomolecular target. Additionaly, we consider compound *B*, which only harbors substructure *S*_2_. Contradictory results would be given by the two methods with respect to the influence of substructure *S*_1_ on bioactivity.

The predictive method would predict a similar bioactivity value for compound *A* with and without substructure *S*_1_, as the bioactivity depends on substructure *S*_2_. By contrast, the Student’s method would consider substructure *S*_1_ as relevant for bioactivity given that the difference between the bioactivities of compounds *A* and *B*, *i.e.* either presenting or not substructure *S*_1_, would be significant. It follows from the preceeding that the predictive method is best suited to give insight into the contribution of single substructures to the bioactivity of individual compounds, whereas the Student’s method is more suited for the identification of the general relevance of the substructures to bioactivity. Another important consideration is the presence of substructures whose effects on bioactivity are correlated. In the situation where a compound presents two substructures whose influences on bioactivity are correlated, the predictive method would likely predict a similar activity when either of them is deleted. Covering diverse structures in the dataset might alleviate this issue, as the probability of finding repeated substructure pairs is likely to decrease with chemical diversity and dataset size. Overall, if the general influence of a substructure on bioactivity is assessed with the predictive method, both the mean value and the standard deviation of the differences between the predicted bioactivity values with and without a given substructure should be reported, as the standard deviation indicates whether the influence of that substructure to bioactivity depends on other substructures or not [68].

In the Student’s method, the pIC_50_ difference associated to a significant p-value might be negligible from a medicinal chemistry standpoint. In addition, the capability of the *t*-test to identify significant differences depends on the sample size. Thus, a small pIC_50_ difference can be detected as significant if the sample size is large, whereas it might not be detected for smaller samples. Therefore, the conclusions extracted from the application of the Student’s method depend on the analyzed dataset, whereas the predictive method might be less dependent on the dataset composition if the models are applied within their applicability domain. In the present study, we have not applied any method to control the family-wise error rate which comes from the multiple comparisons problem [88]. However, we anticipate that in other studies comprising a larger number of substructures, it would be advisable to control this problem. For a recent and detailed discussion of the application of the student *t*-test to assess the statistical significance of bioactivity differences in the context of Matched Molecular Pair Analysis (MMPA), the reader is referred to Kramer *et al.* [89]. In summary, the application of both methods can help to unravel whether the contribution of a given substructure to compound bioactivity depends exclusively on itself, or on the presence of other substructures or chemical scaffolds [90].

## Conclusions

Ensemble modeling has been introduced in the context of PCM to predict the potency of mammalian cyclooxygenase inhibitors. The combination of single models in model ensembles has led to increased predictive ability, as well as to the definition of confidence intervals for individual predictions. PCM has been shown to enable the prediction of the potency for compounds exhibiting uncorrelated bioactivity profiles with high confidence. Finally, the implementation of two different substructure analysis pipelines, which reliability for different purposes has been pointed out, has permitted the recognition of chemical moieties implicated in potency and selectivity in agreement with the medicinal chemistry literature.

## Additional files

Additional file 1
**Data set S1.** The complete dataset used in this study containing: (i) compound descriptors, (ii) amino acid descriptors, and (iii) compound bioactivities.

Additional file 2
**Figure S1.** PCA analysis of the target space. PCA analysis was applied on the binding site descriptors used to train the models. The first two principal components explained more than 80% of the variance, thus indicating that there are mainly two sources of variability in the descriptor space, namely the isoenzyme type. This fact can be seen as COX-1 (triangles) and COX-2 (squares) define two distant clusters. Overall, the binding sites of orthologue cyclooxygenases are more similar than those of paralog sequences. These results also indicate that the amino acid descriptors account for structural differences between COX-1 and COX-2, which can be learnt by the models. Thus, it is expected that merging orthologues and paralogues will lead to more predictive models.

Additional file 3
**Figure S2.** Statistiscs of the repeated bioactivities for each compound-target combination. **A**. The abcissa represents the mean value for the bioactivities repeated for each compound-target combination with more than one annotated bioactivity. The ordinate represents their standard deviations. Repeated bioactivities are equally distributed for low, moderate and high affinity COX inhibitors. **B**. Histogram of the standard deviation of the repeated bioactivities. The distribution is strongly skewed towards 0, thus indicating that the differences between repeated bioactivities are generally negligible.

Additional file 4
**Figure S3.** PCA of the compound descriptors used to train the PCM models. The PCA was performed on the pairwise Pearson rank correlation matrix calculated with the compound descriptors used to train the models. The two first principal components (PC) explain 58.03% of the variance. COX-1 and COX-2 are represented with squares and triangles respectively. Overall, the overlap between the datapoints indicate that the compounds annotated on different targets cover the same regions of the chemical space.

Additional file 5
**Figure S4.** Y-scrambling. Scatterplots corresponding to the percentage of bioactivities randomized, against **(A)** $R^{2}_{0\ test}$ and **(B)** RMSE_test_ values. The intercept in **A** becomes negative when 25-50% of the bioactivity variable is randomized. This finding indicates that PCM performance is not the consequency of spurious correlations in the descriptor space.

Additional file 6
**Figure S5.** Jaccard pairwise similarity distributions for the compounds annotated on each target. Compounds annotated on the human cyclooxygenases (annotated with a star in the plots) display compound similarity distributions with mean values skewed towards 1. By contrast, compounds annotated on targets with less than 30 annotated bioactivities display multimodal similarity distributions. A correlation between model performance and both the number of datapoints and chemical diversity was established (see main text). Distributions were calculated with the same descriptors than the ones used to train the PCM models.

Additional file 7
**Figure S6.** Compound substructures predicted to increase the bioactivity on human COX-2. The 20 substructures predicted to have the highest influence on bioactivity on human COX-2 (P35354) are plotted. Known chemical moieties such as pyrrole rings (1), aryl substituents (*e.g.* 4 and 5) or benzylsulfonamide (17) are represented. These substructures appear in diverse NSAIDs such as rofecoxib or etericoxib, as well as in chemical families of COX-2 inhibitors based on *e.g.* 1,5-diarylpyrazoles or 3,4-diaryl-substituted furans [23-25].

Additional file 8
**Figure S7.** Compound substructures predicted to have the same influence on human COX-1 and COX-2. Substrucutures predicted to decrease bioactivity are accompanied by a blue arrow, whereas that predicted to increase bioactivity are followed by a red arrow. Smaller substructures are found in this case, predominating substituents on the benzene ring. Therefore, substructure-activity relationships are difficult to be determined.
